# Effect of hypoxia-induced mIL15 expression on expansion and memory progenitor stem-like TILs *in vitro*

**DOI:** 10.3389/fimmu.2024.1450245

**Published:** 2024-11-22

**Authors:** Zhen Sun, Aotian Xu, Zhaojun Wu, Xiaohao Lan, Ganchen Gao, Bin Guo, Zhongjie Yu, Lin Shao, Hao Wu, Min Lv, Yongjie Wang, Yi Zhao, Bin Wang

**Affiliations:** ^1^ Department of Pathogenic Biology, School of Basic Medicine, Qingdao University, Qingdao, China; ^2^ Department of Research and Development, Qingdao Sino-Cell Biomed Co., Ltd., Qingdao, Shandong, China; ^3^ Department of Thoracic Surgery, The Affiliated Hospital of Qingdao University, Qingdao, Shandong, China; ^4^ Department of Special Medicine, School of Basic Medicine, Qingdao University, Qingdao, China

**Keywords:** lung cancer, tumor-infiltrating lymphocytes, membrane-bound interleukin 15, expansion, adoptive cell transfer, immunotherapy, hypoxia regulation

## Abstract

**Introduction:**

The adoptive cell transfer of tumor-infiltrating lymphocytes (TILs) has proven clinically beneficial in patients with non-small cell lung cancer refractory to checkpoint blockade immunotherapy, which has prompted interest in TIL-adoptive cell transfer. The transgenic expression of IL15 can promote the expansion, survival, and function of T cells *ex vivo* and *in vivo* and enhance their anti-tumor activity. The effect of expressing mIL15 regulated by hypoxia in the tumor microenvironment on the expansion, survival, and stem-like properties of TILs has not been explored.

**Methods:**

Using TILs expanded from the tumor tissues of lung cancer patients, TILs with or without mIL15 expression (TIL-mIL15 or UN-TIL) were generated by lentiviral transduction. To reflect the advantages of mTIL15, the cells were divided into groups with IL2 (TIL-mIL15+IL2) or without IL2 (TIL-mIL15-IL2).

**Results:**

Compared to UN-TIL cells, mIL15 expression had a similar capacity for promoting TIL proliferation and maintaining cell viability. Our experimental findings indicate that, compared to UN-TIL and TIL-mIL15+IL2 cells, the expression of mIL15 in TIL-mIL15-IL2 cells promoted the formation of stem-like TILs (CD8^+^CD39^-^CD69^-^) and led to significant decreases in the proportion and absolute number of terminally differentiated TILs (CD8^+^CD39^+^CD69^+^). RNA-Seq data revealed that in TIL-mIL15-IL2 cells, the expression of genes related to T cell differentiation and effector function, including *PRDM1*, *ID2, EOMES, IFNG, GZMB*, and *TNF*, were significantly decreased, whereas the expression of the memory stem-like T cell marker *TCF7* was significantly increased. Furthermore, compared to UN-TIL and TIL-mIL15+IL2 cells, TIL-mIL15-IL2 cells showed significantly lower expression levels of inhibitory receptors LAG3, TIGIT, and TIM3, which was consistent with the RNA-Seq results.

**Discussion:**

This study demonstrates the superior persistence of TIL-mIL15-IL2 cells, which may serve as a novel treatment strategy for lung cancer patients.

## 1 Introduction

The combination of different immunotherapy strategies in recent years has contributed to the development of cancer treatment that exploit the effectiveness and potential of the immune system in attacking cancer cells. Such immunotherapy modalities aim to improve the prognosis of cancer patients. These include immune checkpoint inhibitors (CTLA4 and PD-1/PD-L1 axis) (1–4), specific monoclonal antibodies (5), immunostimulants (e.g., BCG) (6), cancer vaccines (7), and the adoptive transfer of tumor-reactive immune cells, known as adoptive cell therapy (ACT) (8). ACT is a personalized strategy that involves infusing an *ex vivo*-expanded pool of endogenous tumor-reactive T cells, such as tumor-infiltrating lymphocyte (TIL) (9) and endogenous T-cell (10), or artificially producing tumor-reactive T cells (11), such as engineered T cells expressing transgenic T-cell receptors (TCRs) or chimeric antigen receptors (CARs) (12). TILs are tumor-specific lymphocytes isolated directly from resected tumors and have shown remarkable clinical efficacy in patients with metastatic melanoma. Reports from several independent centers have consistently shown objective response rates of 40–50%, with complete tumor regression in 10–20% of patients (13–18). TIL products are highly polyclonal; hence, TIL-based ACT benefits from the multi-target T-cell attack against multiple different and mostly unknown antigens (19). Studies have demonstrated that the effectiveness of TIL-based ACT in melanoma patients stems from the specific recognition of neoantigens (20, 21). As with melanomas, the presence of tumor-reactive TILs has also been reported in non-small cell lung cancer (NSCLC), which prompted the exploration of TIL-ACT in NSCLC (22, 23).

Endogenous and administered T cells receive pro-survival signals, such as those mediated by IL2 and IL15, via the common cytokine receptor γ chain, which is independent of innate or adaptive immune receptors. As a T-cell growth factor, IL2 remains the most common cytokine used in the expansion of therapeutic T-cell products for patients (24). Nevertheless, during the process of *ex vivo* expansion, repeated stimulation of T cells with IL2 resulted in T cell exhaustion and reduced T cell persistence (25). Clinically, in patients infused with exogenous IL2, high doses led to dose-limiting toxicity (26), whereas low doses facilitated peripheral tolerance and the production of regulatory T cells (27). Conversely, the pro-survival cytokine IL15 is essential for maintaining the homeostasis of long-lived CD8^+^ memory T cells (28), inhibiting activation-induced cell death (29), and enhancing *in vivo* anti-tumor activity (30). Preclinical studies have found that T cells cultured in IL15 or expressing IL15 showed excellent anti-tumor abilities *in vivo* (30, 31). A major breakthrough came from the observation that elevated IL15 and IL7 levels caused by chemotherapy-induced lymphodepletion prior to ACT can effectively support the function of cell transfer (32, 33).

Although IL2 has been approved by the US FDA, IL15 may offer more advantages in cancer treatment. The phase I trial of IL15 in patients with metastatic melanoma or renal cell cancer reported that five patients manifested a 10–30% decrease in marker lesions, including two patients who experienced clearance of lung lesions (34). Although IL15 has shown remarkable efficacy in the treatment of metastatic malignancies, its rapid renal clearance and biochemical instability may impede its therapeutic potential (34). The IL15 can occur naturally as an IL15Ra/IL15 heterodimeric cytokine in mouse and human serum (35). Heterodimeric IL15 has a longer half-life and stronger bioactivity, which is due to its stronger affinity for the IL15Rβ-γ complex (36). In preclinical trials, IL15 pre-combined with IL15Ra or IL15Ra-IgG1-Fc improved its pharmacokinetics and enhanced its efficacy at increasing the number of circulating natural killer (NK) and CD8^+^ T cells (35). The results of clinical trials showed that administration of the IL15 super-agonist complex ALT-803 enhanced the anti-tumor effect of responsive T cells (37). On April 22, 2024, the IL15 super-agonist N-803 (Anktiva) developed by ImmunityBio was approved by the FDA for use in combination with BCG to treat BCG-unresponsive non-muscle invasive bladder cancer with carcinoma *in situ*.

Approximately50–60% of solid tumors contain hypoxic regions, which are associated with metastasis, resistance to chemotherapy and radiotherapy, and poor patient survival (38, 39). Exploiting this unique environmental signal for targeted therapy is a promising strategy in cancer treatment. To enable cells to sense and respond to hypoxia, several researchers have performed transcriptional and post-translational control on genes of interest using HIF-1α and hypoxia response element (HRE) (40, 41). Collet et al. used an HRE-driven soluble form of VEGFRt2 to control angiogenesis and reduce the growth of hypoxic tumor cells *in vitro* and *in vivo* (42). HRE-IL2-engineered cytotoxic T lymphocytes can produce more rapid and complete tumor regression than parental cytotoxic T lymphocytes, thus increasing the overall survival of tumor-bearing mice (43).

In this study, in order to improve the persistence and memory potential of TILs, we modified TILs using HRE to induce mIL15 expression. The expression of mIL15 can promote the long-term proliferation of TILs and maintain their cell viability. Furthermore, mIL15 expression can promote the generation of memory progenitor stem-like TILs (CD8^+^CD39^-^CD69^-^) and reduce the proportion and absolute number of terminally differentiated TILs (CD8^+^CD39^+^CD69^+^). RNA-Seq data have shown that in TIL-mIL15-IL2 cells, the expression of the memory stem-like T cell marker *TCF7* was increased. Furthermore, TIL-mIL15-IL2 cells showed lower levels of inhibitory receptors LAG3, TIGIT, and TIM3. In summary, mIL15 offers an optimal signaling pathway to prolong the persistence of TILs *in vitro*, thereby providing durable immune surveillance and therapeutic potential.

## 2 Materials and methods

### 2.1 Culture of cell lines

The cell lines used in this study were as follows: HEK293T cells (Embryonic kidney cell line, ATCC, #CRL-3216), Jurkat cells (ATCC, #TIB-152), K562 cells (ATCC, #CCL­243), NCI-H520 cells (Lung Carcinoma, ATCC, #HTB-182), and HCC827 (Lung Carcinoma, ATCC, #CRL-2868). Jurkat, K562, NCI-H520, and HCC827 cells were cultured in RPMI 1640 medium (Servicebio, #G4535) containing 10% fetal bovine serum (FBS; TransGen, #FS301-02) and 1% penicillin-streptomycin (Solarbio, #P1400-100ml). HEK293T cells were cultured in Dulbecco’s Modified Eagle Medium (DMEM, Shanghai Basal Media Technologies, #L110KJ) containing 10% FBS and 1% penicillin-streptomycin. All cells were placed in a humidified incubator at 37°C and 5% CO_2_ for long-term culture.

### 2.2 Construction of lentiviral vector

The lentiviral vector, pLV-EF1a-c-MYC-IRES-EGFP, from Wuhan Miaoling Biotechnology Co., Ltd. was used as the basic framework for construction. The original promoter was replaced with 3× HRE (TGTCACGTCCTGCACGACTCTAGTTGTCACGTCCTGCACGACTCTAGTTGTCACGTCCTGCACGACGCTAGC) and the human thymidine kinase (TK) minimal promoter to construct a lentiviral vector for hypoxia-inducible expression. This lentiviral vector also contained membrane-expressed IL15 (NWVNVISDLKKIEDLIQSMHIDATLYTESDVHPSCKVTAMKCFLLELQVISLESGDASIHDTVENLIILANNSLSSNGNVTESGCKECEELEEKNIKEFLQSFVHIVQMFINTS), the CD86 hinge region (LEDPQPPPDHIP), a transmembrane domain (WITAVLPTVIICVMVFCLILW) and intracellular domain (KWKKKKRPRNSYKCGTNTMEREESEQTKKREKIHIPERSDEAQRVFKSSKTSSCDKSDTCF), as well as hEGFRt (RKVCNGIGIGEFKDSLSINATNIKHFKNCTSISGDLHILPVAFRGDSFTHTPPLDPQELDILKTVKEITGFLLIQAWPENRTDLHAFENLEIIRGRTKQHGQFSLAVVSLNITSLGLRSLKEISDGDVIISGNKNLCYANTINWKKLFGTSGQKTKIISNRGENSCKATGQVCHALCSPEGCWGPEPRDCVSCRNVSRGRECVDKCNLLEGEPREFVENSECIQCHPECLPQAMNITCTGRGPDNCIQCAHYIDGPHCVKTCPAGVMGENNTLVWKYADAGHVCHLCHPNCTYGCTGPGLEGCPTNGPKIPSIATGMVGALLLLLVVALGIGLFM) linked to the polypeptide linker sequence T2A (EGRGSLLTCGDVEENPGP) that can be cleaved *in vivo*.

### 2.3 Production of lentiviral vectors

The lentiviral supernatant was produced as described below. Lentiviral particles were prepared by transiently transfecting HEK293T cells using the Lipo3000 Transfection Kit (Invitrogen, #L3000015). HEK293T cells cultured in a 100-mm tissue culture dish were transfected with 10 μg of the constructed lentiviral vector, 5 μg of the VSV-g envelope plasmid PMD2.G, and 5 μg of the envelope plasmid psPAX2 encoding gag-pol. At 48 h after transfection, the lentiviral supernatant was harvested and filtered through a 0.45 μm filter (Sartorius, #16533-K). The lentiviral particles were concentrated by ultracentrifugation at 28000 rpm for 2 h using the Sorvall LYNX6000 Superspeed Centrifuge (Thermo Fisher). Then, the supernatant was discarded, TIL cell culture medium was added, and the virus was resuspended at 4°C for 2 h and stored at -80°C. The titer of the lentiviral stock was measured using the Jurkat cells.

### 2.4 Lung cancer TILs: culture, lentiviral infection, hypoxia treatment, and phenotype detection

Lung cancer patients were recruited from The Affiliated Hospital of Qingdao University, which was a local hospital in Qingdao, China. This study was approved by the Ethics Committee of The Affiliated Hospital of Qingdao University (approval no.: QYFYKYLL920511921). The lung cancer tumor collected was placed in a 100-mm petri dish and washed with 1× phosphate-buffered saline (PBS; HyClone, SH30256.FS). A pair of sterile ophthalmic scissors or a scalpel was used to remove necrotic areas and connective tissues, and the tumor was cut into small pieces of 1-3 mm^3^ in size. The tumor was then cultured in Advanced RPMI 1640 Medium (Gibco, #12633-012) containing 10% AB serum (Gemini, #100-512-100), 1% L-glutamine (Solarbio, #G0200), 1% penicillin-streptomycin, and 6000 IU/ml human IL2 (Beijing T&L Biotechnology, #TL-104). Cells were observed under microscopy. If no adherent cells were found, the medium was replaced the next day. If a gradual increase in lymphocytes or decrease in cancer cells was not observed, the medium was further replaced. If the lymphocyte increased significantly, such as with the appearance of cell clusters, the cells were passaged and further cultured in an incubator at 37°C and 5% CO_2_. Culturing was continued for no more than 10 days, and the TILs harvested served as the pre-REP (Tumor Fragment Culture) TILs. For the transduction of pre-REP TILs, lentiviral particles (MOI=10) and TILs activated by anti-hCD3/hCD28 (Beijing Sino Biological Inc.) were incubated for 24 h in REP medium containing 1× Lentiboost (SIRIONBiotech, #SB-A-LF-901-01) and 6000 IU/ml human IL2. The next day, the culture medium was replaced with fresh REP medium. At 5 day after TIL transduction, the Hypoxia Incubator Chamber (Stemcell, #27310) was used to adjust the oxygen concentration to less than 1%, and hypoxia treatment was performed for 24 h. The expression of mIL15 and hEGFRt were measured using flow cytometry as described below.

### 2.5 Preparation of feeder cells

#### 2.5.1 Irradiated peripheral blood mononuclear cells

Leukocytes obtained from apheresis were purchased from Shanghai Milestone Biotechnologies and irradiated at an intensity of 50 Gy. After 1 h of irradiation, PBMCs were extracted from irradiated leukocytes using density gradient centrifugation, and stored in a liquid nitrogen container at a cell density of 1–2× 10^8^ cells/ml for later use.

#### 2.5.2 K562-mIL21-4-1BBL cells

Gene synthesis was performed which contained the sequences for 4-1BBL (MEYASDASLDPEAPWPPAPRARACRVLPWALVAGLLLLLLLAAACAVFLACPWAVSGARASPGSAASPRLREGPELSPDDPAGLLDLRQGMFAQLVAQNVLLIDGPLSWYSDPGLAGVSLTGGLSYKEDTKELVVAKAGVYYVFFQLELRRVVAGEGSGSVSLALHLQPLRSAAGAAALALTVDLPPASSEARNSAFGFQGRLLHLSAGQRLGVHLHTEARARHAWQLTQGATVLGLFRVTPEIPAGLPSPRSEGACRA), mIL21 (MRSSPGNMERIVICLMVIFLGTLVHKSSSQGQDRHMIRMRQLIDIVDQLKNYVNDLVPEFLPAPEDVETNCEWSAFSCFQKAQLKSANTGNNERIINVSIKKLKRKPPSTNAGRRQKHRLTCPSCDSYEKKPPKEFLERFKSLLQKMIHQHLSSRTHGSEDS), IRES, mCherry (MVSKGEEDNMAIIKEFMRFKVHMEGSVNGHEFEIEGEGEGRPYEGTQTAKLKVTKGGPLPFAWDILSPQFMYGSKAYVKHPADIPDYLKLSFPEGFKWERVMNFEDGGVVTVTQDSSLQDGEFIYKVKLRGTNFPSDGPVMQKKTMGWEASSERMYPEDGALKGEIKQRLKLKDGGHYDAEVKTTYKAKKPVQLPGAYNVNIKLDITSHNEDYTIVEQYERAEGRHSTGGMDELYK) and Puro (MTEYKPTVRLATRDDVPRAVRTLAAAFADYPATRHTVDPDRHIERVTELQELFLTRVGLDIGKVWVADDGAAVAVWTTPESVEAGAVFAEIGPRMAELSGSRLAAQQQMEGLLAPHRPKEPAWFLATVGVSPDHQGKGLGSAVVLPGVEAAERAGVPAFLETSAPRNLPFYERLGFTVTADVEVPEGPRTWCMTRKPGA) (Nanjing GenScript Biotech). The sequences were then cloned into the lentiviral vector pLV-EF1a-c-MYC-IRES-EGFP, replacing the original c-MYC-IRES-EGFP sequence. Once the plasmid was obtained, lentiviral packaging was performed and its titer was calculated. K562 cells were infected at MOI=10. Lentivirus-infected K562 cells were treated with 2 µg/ml puromycin (Solarbio, #P8230) for 48 h, followed by 6 µg/ml mitomycin C (MCE, #HY-13316) for 24 h to obtain K562-mIL21-4-1BBL cells.

### 2.6 Expansion of TILs

1× 10^5^ pre-REP TILs or transduced TILs were co-incubated with 2.5× 10^6^ irradiated PBMCs or K562-mIL21-4-1BBL cells in REP medium containing 30 ng/ml anti-hCD3 (Beijing T&L Biotechnology, #GMP-TL101). Human IL2 was added according to the experimental design. Half of the culture medium was replaced every three days with fresh medium without anti-hCD3. At 14 day, the viability of TILs was analyzed by staining with acridine orange/propidium iodide (AO/PI, CountStar, #RE010212) and harvested as REP (rapid expansion) TILs.

### 2.7 Flow cytometry analysis

For flow cytometry, cell surface staining was performed using the following antibodies: anti-human CD3 (BioLegend, #317332), anti-human CD8 (BioLegend, #344714), anti-human CD4 (BioLegend, #317429), anti-human CD39 (BioLegend, #328208), anti-human CD69 (BioLegend, #310910), anti-human CD279 (PD-1) (BioLegend, #621608), anti-human CD366 (TIM3) (BioLegend, #345012), anti-human TIGIT (BioLegend, #372706), anti-human 4-1BBL (BioLegend, #311506), anti-human IL21 (BD Biosciences, #562043) and anti-human TCF1 (BioLegend, #655208). For the detection of mIL15 and hEGFRt, cells were incubated in IL15 (Beijing BioNC Biotech) and cetuximab (Merck) antibody for 15 min, washed twice, and then stained with FITC Goat anti-human IgG Fc γ Antibody (BioLegend, #398006) and Alexa Fluor 647 Donkey anti-rabbit IgG Antibody (BioLegend, #4064) at room temperature for 10 min. Staining with 7-AAD (BioLegend, #420404) was performed to identify dead cells. For detection of TCF1, surface-stained cells were fixed and permeabilized with the True-Nuclear Transcription Factor Buffer Set (BioLegend, #424401), followed by incubation with anti-human TCF1 antibody. Flow cytometry data were collected on a CytoFLEX (Beckman-Coulter, Fullerton, CA, USA) and were analyzed with FlowJo software (TreeStar).

### 2.8 Cytokine release assay

For the cytokine release assay, 1× 10^6^ UN-TIL+IL2, TIL-mIL15+IL2 and TIL-mIL15-IL2 cells were co-incubated separately with 5× 10^5^ human T Cell Activation/Expansion magnetic beads (Miltenyi Biotec, Germany, #130-091-441) at 37°C for 24 h. The supernatant was collected, and the concentrations of IFNγ (Dakewe, #1110002) and GZMB (Dakewe, #1118502) were measured using enzyme-linked immunosorbent assay (ELISA).

### 2.9 Killing assay

NCI-H520 and HCC827 cells were labeled with mCherry using a lentiviral infection platform. NCI-H520-mCherry and HCC827-mCherry cells were seeded to a 24 well plate at a cell density of 8× 10^4^ cells/ml. 24 h later, UN-TIL+IL2, TIL-mIL15+IL2 and TIL-mIL15-IL2 cells were added to tumor cells according to TIL-to-target ratio of 5:1. After 24 h, the killing of tumor cells was observed and recorded under a fluorescence microscope (OLYMPUS).

### 2.10 Preparation of human NK cells

We added 1× PBS containing 15 μg/ml CD16 (Beijing T&L Biotechnology, #GMP-TL201) to the T75 culture flask and incubated in the dark at 37°C for 2 h. After which, 10 ml of 1× PBS was added to wash the culture flask. According to the cell inoculation density of 2.0× 10^6^ cells/ml, PBMCs were centrifuged and the supernatant was discarded. An appropriate amount of NK complete culture medium was added, including RPMI1640 medium, 10% autologous plasma, and 800 IU/ml IL2 (#GMP-TL906), 35 ng/ml IL21 (#GMP-TL509), 50 ng/ml IL15 (#GMP-TL202), 50 ng/ml IL18 (#GMP-TL203), and 40 ng/ml IL12 (#GMP-TL508). Then, the cells were resuspended and slowly added to the CD16-coated T75 flask for culturing. The medium was replaced every two days, and the cells were harvested after culturing for 13–16 days. All cytokines used were purchased from Beijing Tongli Haiyuan.

### 2.11 Antibody-dependent cellular cytotoxicity

1× 10^6^ UN-TIL+IL2 and TIL-mIL15+IL2 cells were incubated with cetuximab (Merck) or rituximab (Merck) at 37°C for 30 min. Human primary NK cells were added at an effector:target ratio (E:T) of 10:1 and incubated at 37°C for 24 h. Then, the cells were harvested for flow cytometry to measure the proportion of mIL15^+^hEGFRt^+^ cells.

### 2.12 Complement-dependent cytotoxicity

1× 10^6^ UN-TIL and TIL-mIL15 cells were resuspended in serum-free medium and incubated with cetuximab or rituximab at 37°C for 30 min. Baby rabbit complement (Cedarlane, #CL3441-S-R) was added, mixed evenly, and further incubated for 4 h. Then, the cells were harvested for flow cytometry to measure the proportion of mIL15^+^hEGFRt^+^ cells.

### 2.13 RNA-seq analysis

Based on the K562-mIL21-4-1BBL cell culture platform, the cells (5× 10^6^ UN-TIL+IL2, TIL-mIL15+IL2, and TIL-mIL15-IL2) were cultured and harvested for transcriptome sequencing to analyze the differences in gene expression. Total RNA was extracted using the EASYspin Plus Tissue/Cell RNA Rapid Isolation Kit (Beijing Aidlab Biotechnologies, #RN2802) according to the manufacturer’s instructions. The Illumina NEBNext^®^ UltraTM RNA Library Prep Kit was used to construct the cDNA library. The insert size of the cDNA library was measured using the Agilent 2100 Bioanalyzer. Once the expected insert size was attained, qRT-PCR was carried out to accurately quantify the effective concentration of the cDNA library, followed by Illumina sequencing. For data analysis, the reads per kilobase per million mapped reads (RPKM) were calculated, differentially expressed genes (DEGs) were defined as log2 (fold change) > 1.5 or log2 (fold change) < -1.5, and edgeR was statistically significant (p<0.05). The datasets generated in this study will be submitted to the GEO.

### 2.14 Data analysis

The statistical significance of differences between groups was determined using two-tailed, unpaired Student’s t-test and GraphPad Prism software (version 8.0). Differences with p-values≥ 0.05 were considered non-significant (NS). p-values<0.05 were considered statistically significant (*P<0.05; **P<0.01; ***P<0.001; ****P<0.0001).

## 3 Results

### 3.1 Generation and identification of mIL15-expression TILs

**Figure 1:**
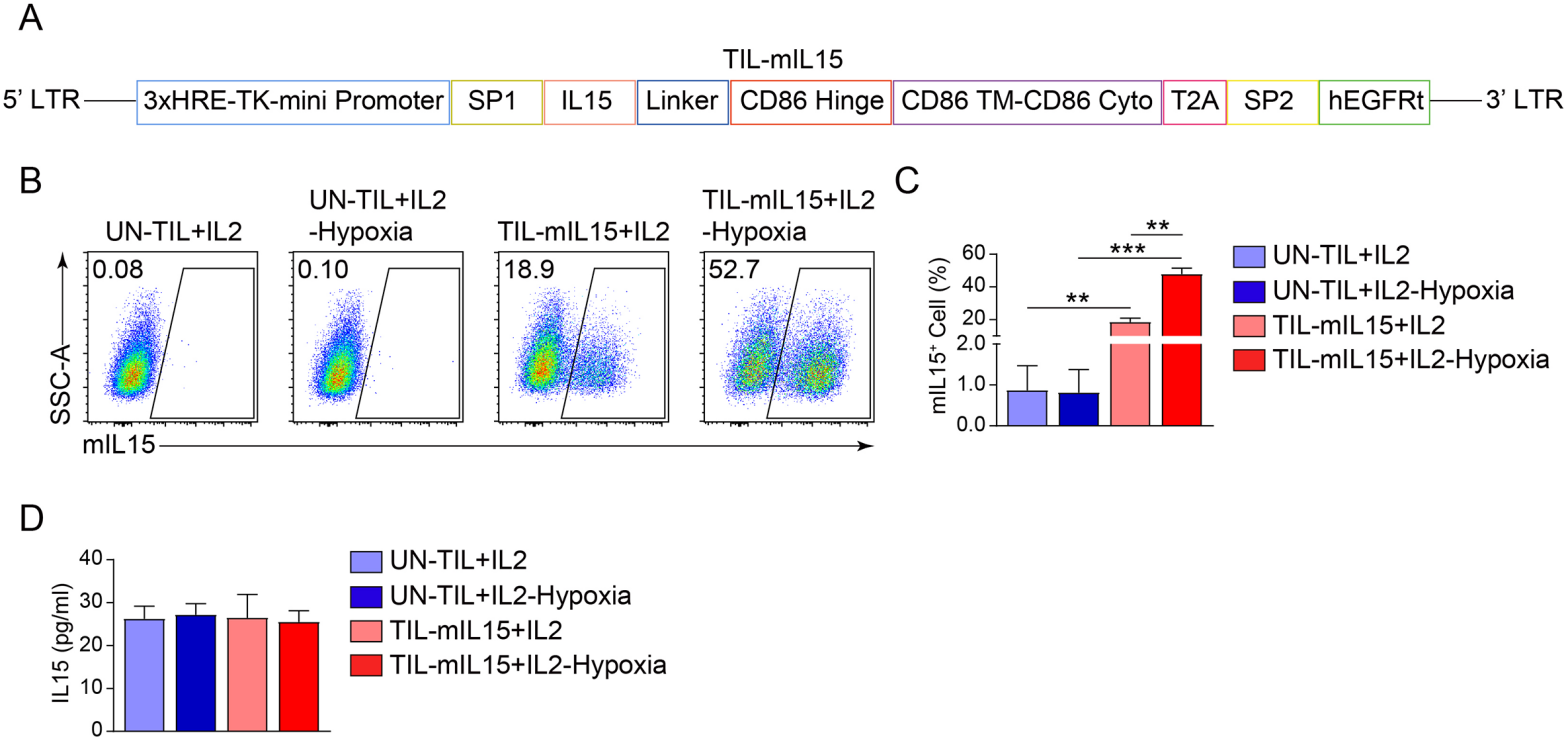
Generation and identification of mIL15-expressing TILs. **(A)** Schematic diagram of lentiviral vector encoding mIL15. **(B)** Representative flow cytometry plot of mIL15^+^ cells at 5 d after pre-REP TIL transduction. **(C)** Statistical plot showing the percentage of mIL15^+^ cells (n=3). **(D)** ELISA analysis of IL15 secretion in culture supernatants of UN-TIL+IL2 and TIL-mIL15+IL2 cells (n=3). Statistical analysis performed using two-tailed unpaired Student’s t-test. Statistical significance defined as: **P<0.01; ***P<0.001.

Reportedly, the IL2 signaling pathway can drive effector T cell proliferation and promote terminal differentiation and senescence (44). In contrast, IL15 is a pro-survival cytokine that is necessary for maintaining the homeostasis of long-lived CD8^+^ memory T cells (28). In order to assess whether IL15 can overcome the clinical shortcomings of IL2, we constructed a mIL15-expressing lentiviral vector (**Figure 1A**). Based on the characteristics of tumor hypoxia (45, 46), the tandem repeat sequence of HREs can drive gene transcription under hypoxic conditions (47). Lentiviral vector was constructed using 3× HRE and the TK minimal promoter to induce the expression of mIL15 under hypoxic conditions. The structure also contains the CD86 hinge region and a transmembrane domain (**Figure 1A**). In addition, the hEGFRt molecule was added to the lentiviral vector to enhance the clinical safety of TIL-mIL15 (**Figure 1A**). To generate TIL-mIL15 cells, pre-REP TILs were activated with the aCD3/CD28 antibody and transduced with the lentivirus encoding mIL15 for 5 days; UN-TIL cells were used as control. As shown in **Figures 1B, C**, TIL-mIL15 cells subjected to 24 h of hypoxia treatment exhibited a higher proportion of mIL15^+^ cells compared to untreated TIL-mIL15 cells. The ELISA results indicated that there was no difference in the amount of IL15 secreted in the culture supernatants of UN-TIL and TIL-mIL15 cells with and without hypoxia treatment, which implies that there was no shedding of IL15 in TIL-mIL15 cells (**Figure 1D**). Consequently, TIL-mIL15 cells were successfully produced for subsequent research.

### 3.2 mIL15 promotes TIL proliferation and maintains cell viability in absence of IL2

**Figure 2:**
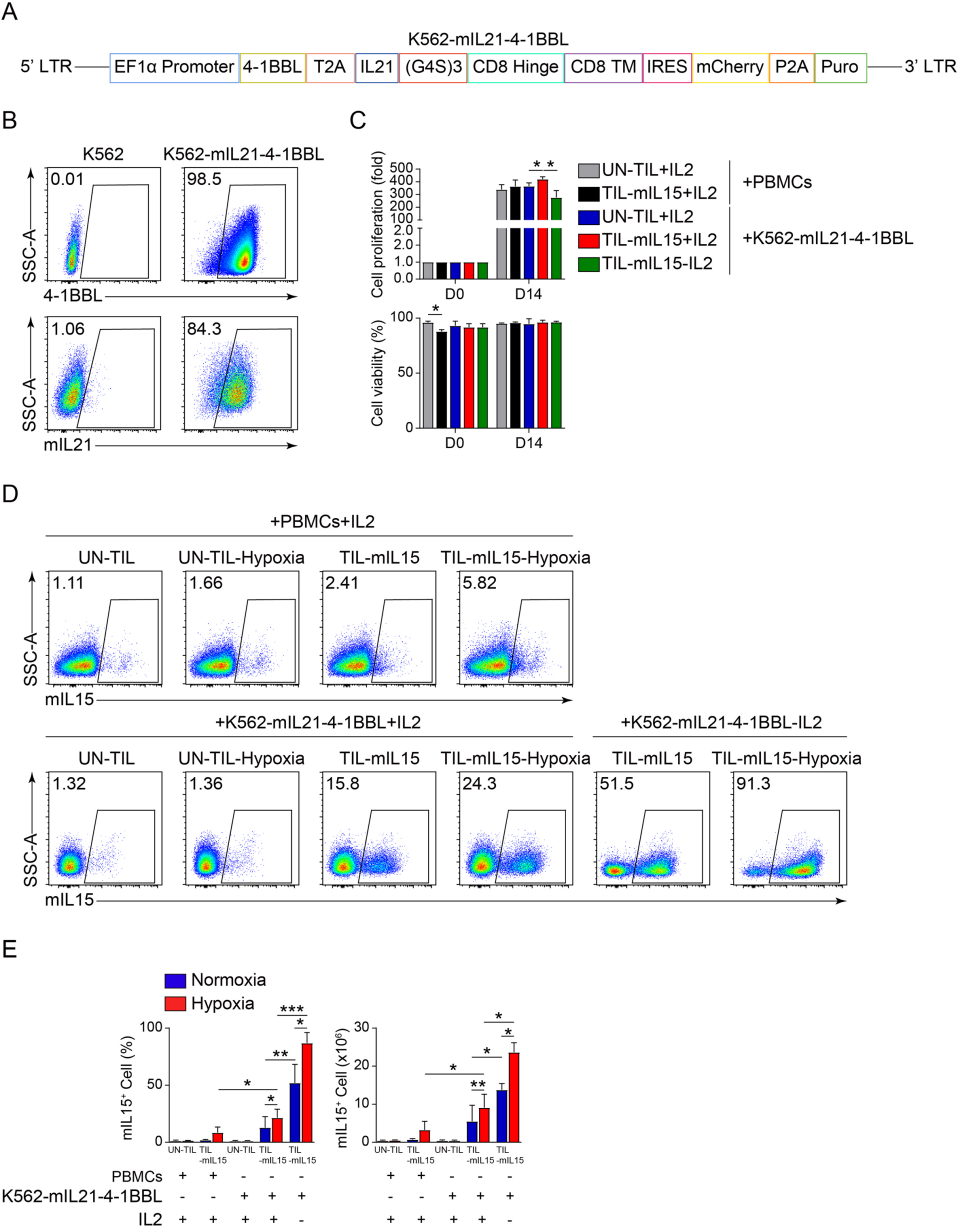
mIL15 promotes TIL proliferation. **(A)** Schematic diagram of lentiviral vector for K562-mIL21-4-1BBL cells. **(B)** Flow cytometry analysis of 4-1BBL and mIL21 expression levels in K562 cells. **(C)** Cell proliferation and viability of UN-TIL+IL2, TIL-mIL15+IL2, and TIL-mIL15-IL2 cells after 14 d of expansion cultured with PBMCs treated with 50 Gy irradiation and K562-mIL21-4-1BBL cells treated with 6 μg/ml mitomycin C as feeder cells (n=3). **(D)** Representative flow cytometry plots of mIL15^+^ cells among UN-TIL+IL2, TIL-mIL15+IL2, and TIL-mIL15-IL2 cells after 14 d of expansion and treatment with hypoxia. **(E)** Statistical plot showing percentage of mIL15^+^ cells (n=3). Statistical analysis performed using two-tailed unpaired Student’s t-test. Statistical significance defined as: *P<0.05; **P<0.01; ***P<0.001.

To examine the effect of mIL15 on TIL proliferation, we used two types of feeder cells: irradiated PBMCs and K562-mIL21-4-1BBL cells. First, we constructed K562-mIL21-4-1BBL cells capable of expressing 4-1BBL and mIL21. The construction of its lentiviral vector is illustrated in **Figure 2A**. The flow cytometry plot in **Figure 2B** showed that 4-1BBL and mIL21 were highly expressed by the K562-mIL21-4-1BBL cells prepared. Next, the pre-REP TILs were transduced with lentivirus encoding mIL15 for 5 days and then co-cultured with irradiated PBMCs or K562-mIL21-4-1BBL cells for 14 days. Their cell proliferation and viability were recorded. After co-culturing for 14 days, the TIL-mIL15+IL2 and UN-TIL+IL2 groups co-cultured with irradiated PBMCs exhibited no difference in cell proliferation or viability (**Figure 2C**). Among the groups co-cultured with K562-mIL21-4-1BBL cells, the TIL-mIL15+IL2 group showed greater cell proliferation than the UN-TIL+IL2 and TIL-mIL15-IL2 groups; no significant difference in cell viability was found among the three groups (**Figure 2C**). Meanwhile, we found that the addition of IL2 affected the expression of mIL15 during the expansion of TIL-mIL15 cells. In the irradiated PBMCs group, the mean percentage of mIL15^+^ cells among TIL-mIL15+IL2 cells treated with hypoxia was about 5%. In the K562-mIL21-4-1BBL group, the mean percentage of mIL15^+^ cells among TIL-mIL15+IL2 cells treated with hypoxia was about 25%, whereas the mean percentage mIL15^+^ cells among TIL-mIL15-IL2 cells treated with hypoxia was about 90%, and the absolute number was higher than that of the TIL-mIL15+IL2 group (**Figures 2D, E**). According to the mIL15 expression level, the K562-mIL21-4-1BBL cell culture platform was superior to irradiated PBMCs. Based on the K562-mIL21-4-1BBL cell culture platform, our findings indicated that mIL15 could replace IL2 to promote proliferation and maintain the viability of TILs. Furthermore, mIL15 was highly expressed by TIL-mIL15-IL2 cells under hypoxia condition.

### 3.3 mIL15 promotes generation of memory progenitor stem-like TILs

**Figure 3:**
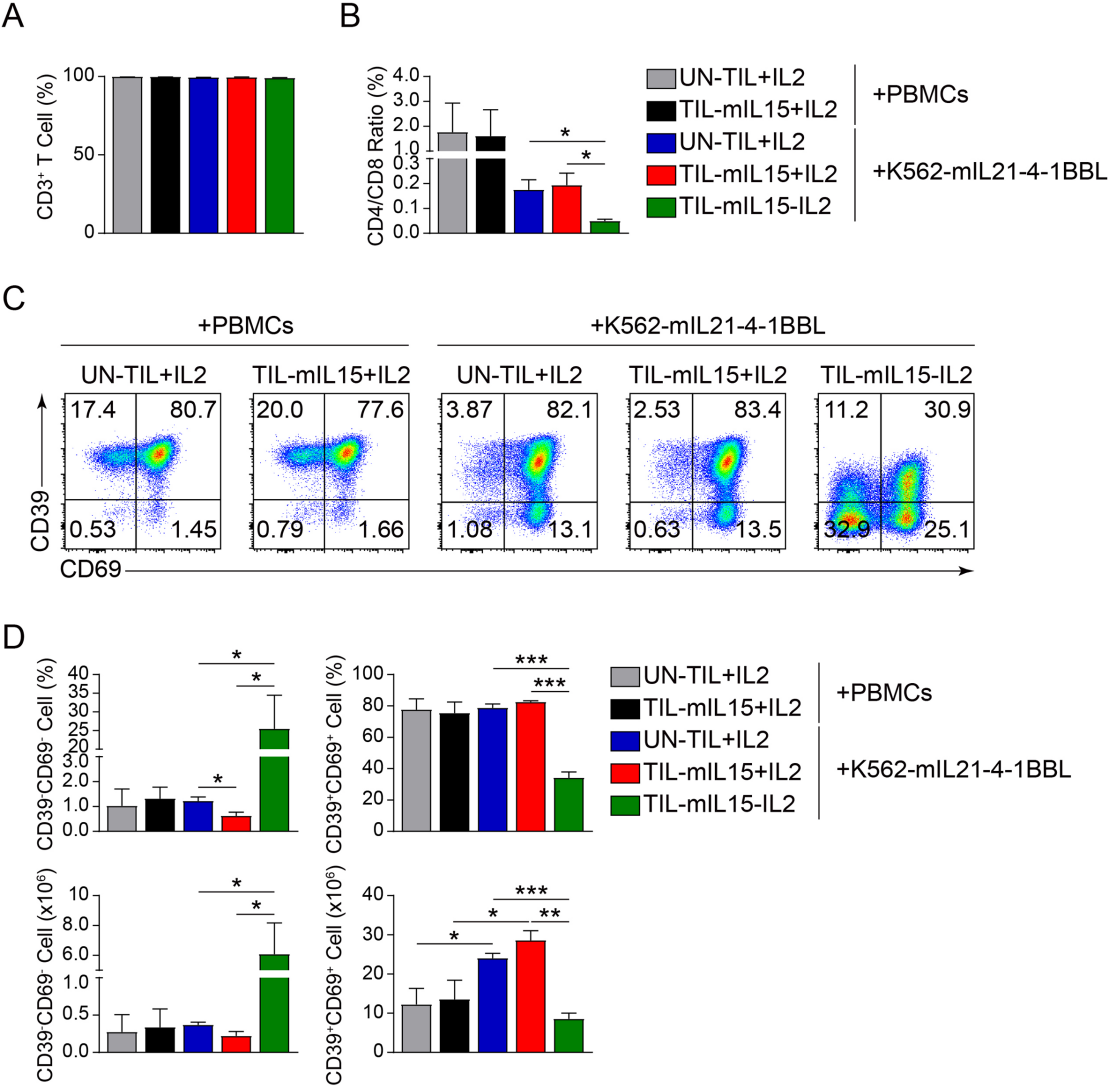
mIL15 promotes the formation of CD8^+^CD39^-^CD69^-^ TILs. **(A)** Statistical plots showing the proportion of CD3^+^ T cells among UN-TIL+IL2, TIL-mIL15+IL2, and TIL-mIL15-IL2 cells. **(B)** Statistical plots showing the CD4/CD8 ratio among UN-TIL+IL2, TIL-mIL15+IL2, and TIL-mIL15-IL2 cells. **(C)** Representative flow cytometry plots of CD39^-^CD69^-^ and CD39^+^CD69^+^ cells among CD8^+^ T cells in the UN-TIL+IL2, TIL-mIL15+IL2, and TIL-mIL15-IL2 groups cultured on the irradiated PBMCs and K562-mIL21-4-1BBL cell culture platforms. **(D)** Statistical plots showing proportion and absolute number of CD39^-^CD69^-^ and CD39^+^CD69^+^ cells among CD8^+^ T cells in the UN-TIL+IL2, TIL-mIL15+IL2, and TIL-mIL15-IL2 groups (n=3). Statistical analysis performed using two-tailed unpaired Student’s t-test. Statistical significance defined as: *P<0.05; **P<0.01; ***P<0.001.

Subsequently, we analyzed the effect of mIL15 on T cell subsets. Based on the irradiated PBMCs and K562-mIL21-4-1BBL cell culture platforms, after 14 days of expansion, the proportion of CD3^+^ T cells was >90% in the UN-TIL+IL2, TIL-mIL15+IL2 and TIL-mIL15-IL2 cells (**Figure 3A**). As shown in **Figure 3B**, based on the irradiated PBMCs cell culture platform, after 14 days of expansion, the CD4/CD8 ratio of the UN-TIL+IL2 and TIL-mIL15+IL2 cells was nearly 2:1, which implies that the proportion of CD4^+^ T cells was higher than that of CD8^+^ T cells. Based on the K562-mIL21-4-1BBL cell culture platform, after 14 days of expansion, the CD4/CD8 ratio of the UN-TIL+IL2 and TIL-mIL15+IL2 cells was <2:1, while the CD4/CD8 ratio of the TIL-mIL15-IL2 cells was significantly lower without the addition of IL2 than with; hence, this condition can facilitate the enrichment of CD8^+^ T cells (**Figure 3B**).

Steven A. Rosenberg’s team performed the high-dimensional analysis of human ACT products and found that CD39^-^CD69^-^ cells exhibited a memory progenitor stem-like phenotype, which was associated with cancer regression and TIL persistence (48). In contrast, CD39^+^CD69^+^ cells showed a terminally differentiated state and were associated with poor TIL persistence (48). Therefore, we further analyzed the effect of mIL15 on the generation of CD8^+^CD39^-^CD69^-^ and CD8^+^CD39^+^CD69^+^ TILs. Based on the irradiated PBMCs and K562-mIL21-4-1BBL cell culture platforms, after 14 days of expansion, compared with UN-TIL+IL2 cells, there was no significant difference in the proportions of CD39^-^CD69^-^ and CD39^+^CD69^+^ cells among CD8^+^ T cells in the TIL-mIL15+IL2 cells (**Figures 3C, D**). Owing to differences in TIL cell counts, there were differences in the absolute number of CD39^+^CD69^+^ cells (**Figures 3C, D**). Based on the K562-mIL21-4-1BBL cell culture platform, compared to the UN-TIL+IL2 and TIL-mIL15+IL2 groups, the proportion and absolute number of CD39^-^CD69^-^ cells among CD8^+^ T cells in the TIL-mIL15-IL2 group were significantly higher, whereas the proportion and absolute number of CD39^+^CD69^+^ cells were significantly lower (**Figures 3C, D**). These results demonstrated that mIL15 expression can promote the generation of memory progenitor stem-like TILs while also reducing the proportion and absolute number of terminally differentiated TILs.

### 3.4 TIL-mIL15 cells exhibit a phenotype of low differentiation and inhibitory receptor expression

In order to confirm the effects of mIL15 on the differentiation, exhaustion, and apoptosis of TILs, we performed transcriptome sequencing and flow cytometry analysis on UN-TIL+IL2, TIL-mIL15+IL2, and TIL-mIL15-IL2 cells subjected to 14 days of expansion on the K562-mIL21-4-1BBL cell culture platform.

**Figure 4:**
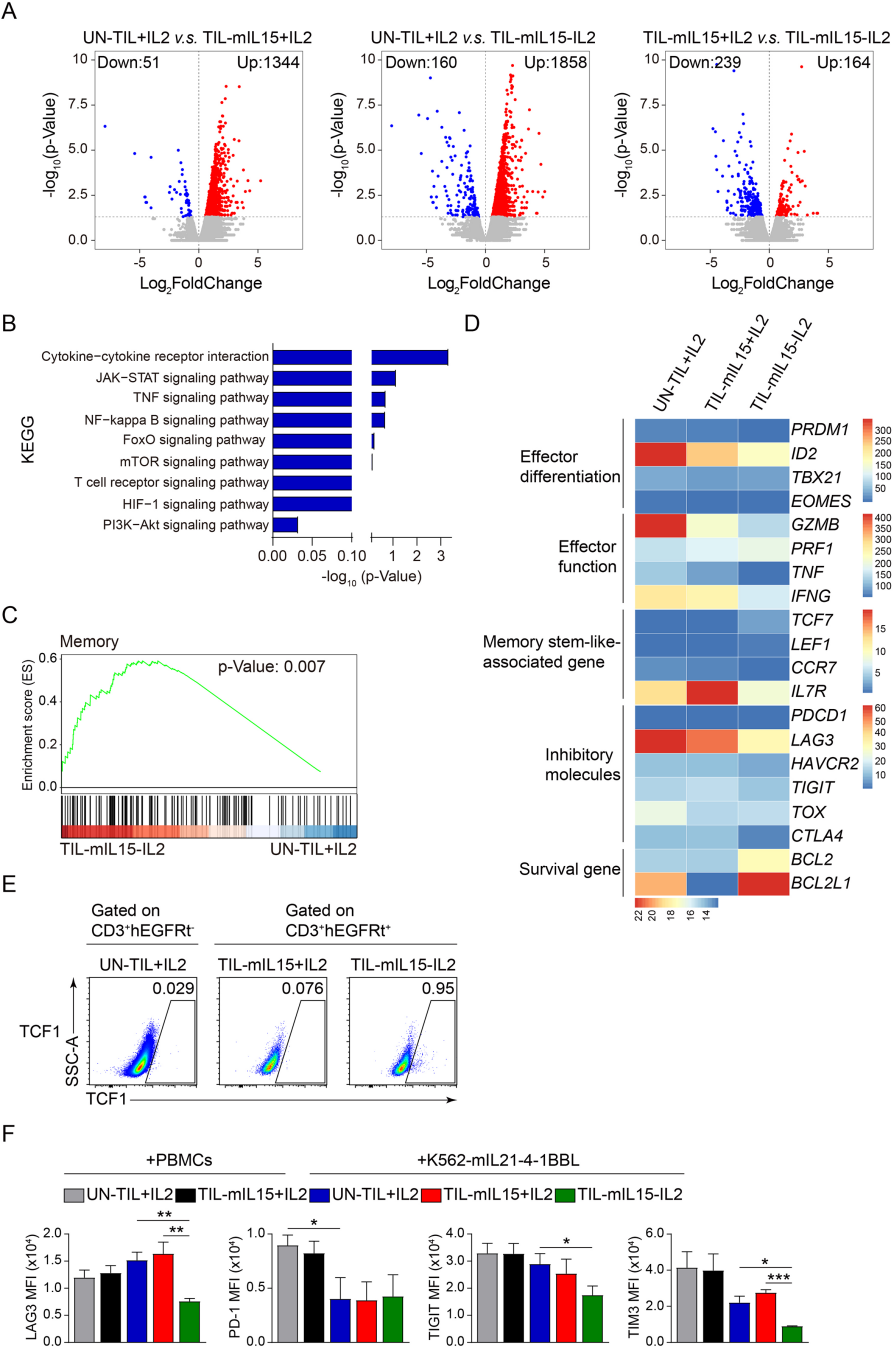
Gene and signal transduction characteristics of TIL-mIL15-IL2 cells. **(A)** RNA-Seq analysis of UN-TIL+IL2, TIL-mIL15+IL2, and TIL-mIL15-IL2 cells after 14 d of expansion on the K562-mIL21-4-1BBL cell culture platform. Volcano plots showing upregulated (red) or downregulated (blue) genes in TIL-mIL15-IL2 cells compared to UN-TIL+IL2 and TIL-mIL15+IL2 cells. **(B)** KEGG pathway analysis of DEGs in TIL-mIL15-IL2 cells relative to UN-TIL+IL2 cells. **(C)** GSEA analysis of gene signatures in TIL-mIL15-IL2 cells relative to UN-TIL+IL2 cells. **(D)** Heatmaps of effector differentiation, effector function, memory stem-like-associated genes, inhibitory molecules, and survival genes. **(E)** Flow cytometry analysis of TCF1 level on CD3^+^hEGFRt^-^ and CD3^+^hEGFRt^+^ cells in the UN-TIL+IL2, TIL-mIL15+IL2, and TIL-mIL15-IL2 cells after 14 d of expansion on K562-mIL21-4-1BBL cell culture platform. **(F)** Statistical plots showing the mean fluorescence intensity (MFI) of inhibitory receptors LAG3, PD-1, TIGIT, and TIM3 among CD8+ T cells in the UN-TIL+IL2, TIL-mIL15+IL2 and TIL-mIL15-IL2 groups after 14 d of expansion on irradiated PBMCs and K562-mIL21-4-1BBL cell culture platforms (n=3). Statistical analysis performed using two-tailed unpaired Student’s t-test. Statistical significance defined as: *P<0.05; **P<0.01; ***P<0.001.

Through RNA-seq analysis, compared with UN-TIL+IL2 cells, TIL-mIL15+IL2 cells showed 1,344 upregulated genes and 51 downregulated genes, while TIL-mIL15-IL2 cells showed 1,858 upregulated genes and 160 downregulated genes; compared with TIL-mIL15+IL2 cells, TIL-mIL15-IL2 cells showed 164 upregulated genes and 239 downregulated genes (**Figure 4A**). In addition, various signaling pathways were enriched in TIL-mIL15-IL2 cells, of which the JAK-STAT, mTOR, and PI3K-Akt pathways were closely associated with the regulation of mIL15 (**Figure 4B**). Characteristic genes related to memory were enriched in TIL-mIL15-IL2 cells (**Figure 4C**). We further selected some differentially expressed genes (DEGs) of interest from among the RNA-Seq results. Our findings revealed that in TIL-mIL15-IL2 cells, the expression of genes related to T cell differentiation and effector function, including *PRDM1* (encoding BLIMP-1), *ID2, EOMES, IFNG, GZMB*, and *TNF*, were significantly decreased, whereas the expression of the memory stem-like T cell marker *TCF7* (encoding TCF1) was significantly increased (**Figure 4D**). Moreover, expression level of TCF1 was much higher in TIL-mIL15-IL2 cells than that of UN-TIL+IL2 and TIL-mIL15+IL2 cells after 14 days of expansion on K562-mIL21-4-1BBL cell culture platform (**Figure 4E**). Furthermore, the expression of pro-survival genes *BCL2* and *BCL2L1* were significantly increased (**Figure 4D**). Additionally, we found that the expression of genes related to inhibitory regulators, including *LAG3*, *HAVCR2* (encoding TIM3), *TIGIT*, *TOX*, and *CTLA4*, were decreased in TIL-mIL15-IL2 cells (**Figure 4D**).

T-cell exhaustion is a dysfunctional state that involves the upregulation of inhibitory receptors (e.g., PD-1, LAG3, and 2B4) in the early to middle stages, accompanied by reduced anti-tumor function (49). T-cell exhaustion can affect the function and persistence of T cells. Hence, methods that can overcome or prevent T cell exhaustion will improve the effectiveness of T cell therapy. Our study revealed that there were no differences in the expression levels of inhibitory receptors LAG3, TIGIT, and TIM3 among CD8^+^ T cells in the TIL-mIL15+IL2 group compared to the UN-TIL+IL2 group after 14 days of expansion on the irradiated PBMCs and K562-mIL21-4-1BBL cell culture platforms (**Figure 4F**, **Supplementary Figure 1**). Compared to the UN-TIL+IL2 and TIL-mIL15+IL2 groups, CD8^+^ T cells in the TIL-mIL15-IL2 group showed significantly decreased expression levels of LAG3 and TIM3, but no difference in the expression level of PD-1 after 14 days of expansion on the K562-mIL21-4-1BBL cell culture platform (**Figure 4F**, **Supplementary Figure 1**). In summary, when cultured in the absence of IL2, mIL15 was able to maintain the poorly differentiated and low exhaustion phenotype of TILs.

### 3.5 mIL15 expression in TILs leads to reduced INF-γ and GZMB secretion

**Figure 5:**
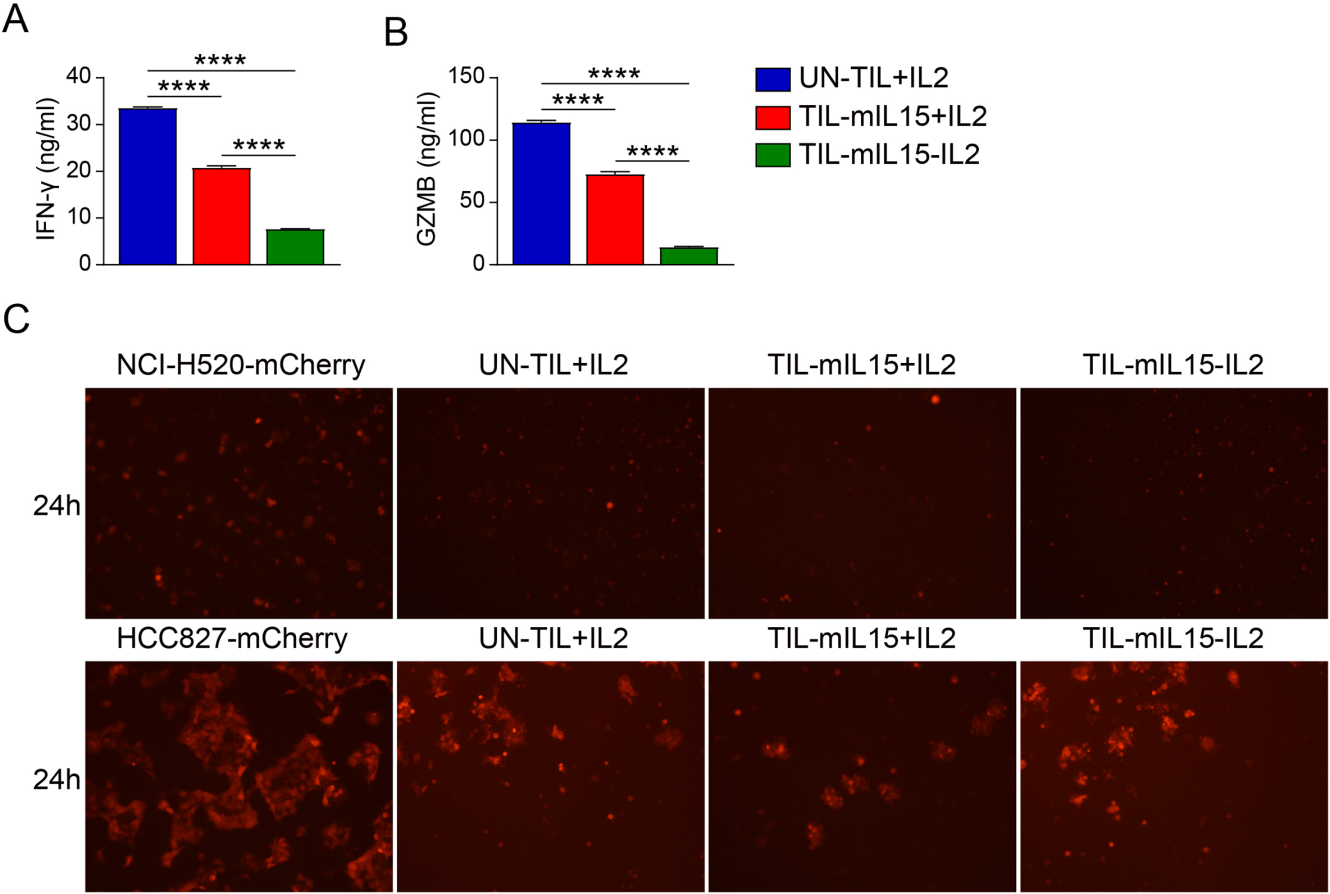
mIL15 regulates the secretion of the antitumor cytokines IFN-γ and GZMB. UN-TIL+IL2, TIL-mIL15+IL2, and TIL-mIL15-IL2 cells were co-incubated with anti-CD2/CD3/CD28-conjugated T cell activation magnetic beads (cells: beads = 2:1) for 24 h, and cell culture supernatant was collected. **(A)** ELISA analysis of IFN-γ level in cell culture supernatant (n=3). **(B)** ELISA analysis of GZMB level in cell culture supernatant (n=3). **(C)** Killing assay (TIL-to-target ratio, 5:1) of UN-TIL+IL2, TIL-mIL15+IL2, and TIL-mIL15-IL2 cells using HLA-matched NCI-H520 (top) and HCC827 (bottom) cells. Statistical analysis performed using two-tailed unpaired Student’s t-test. Statistical significance defined as follows: ****P<0.0001.

In order to evaluate the effect of mIL15 on the production of anti-tumor cytokines, we co-incubated UN-TIL+IL2, TIL-mIL15+IL2, and TIL-mIL15-IL2 cells with anti-CD2/CD3/CD8-conjugated T cell activation magnetic beads and measured the levels of IFN-γ and GZMB in the cell culture supernatant. After 14 days of expansion on the K562-mIL21-4-1BBL cell culture platform, TIL-mIL15+IL2 cells exhibited significantly lower IFN-γ secretion compared to UN-TIL+IL2 cells; TIL-mIL15-IL2 cells showed significantly lower IFN-γ secretion compared to UN-TIL+IL2 and TIL-mIL15+IL2 cells (**Figure 5A**). In addition, we measured the level of GZMB in the cell culture supernatant, and the results were consistent (**Figure 5B**). The killing capacity of TIL-mIL15+IL2 and TIL-mIL15-IL2 cells was similar with UN-TIL+IL2 cells (**Figure 5C**).

### 3.6 TIL-mIL15 cells express hEGFRt to improve its clinical safety

**Figure 6:**
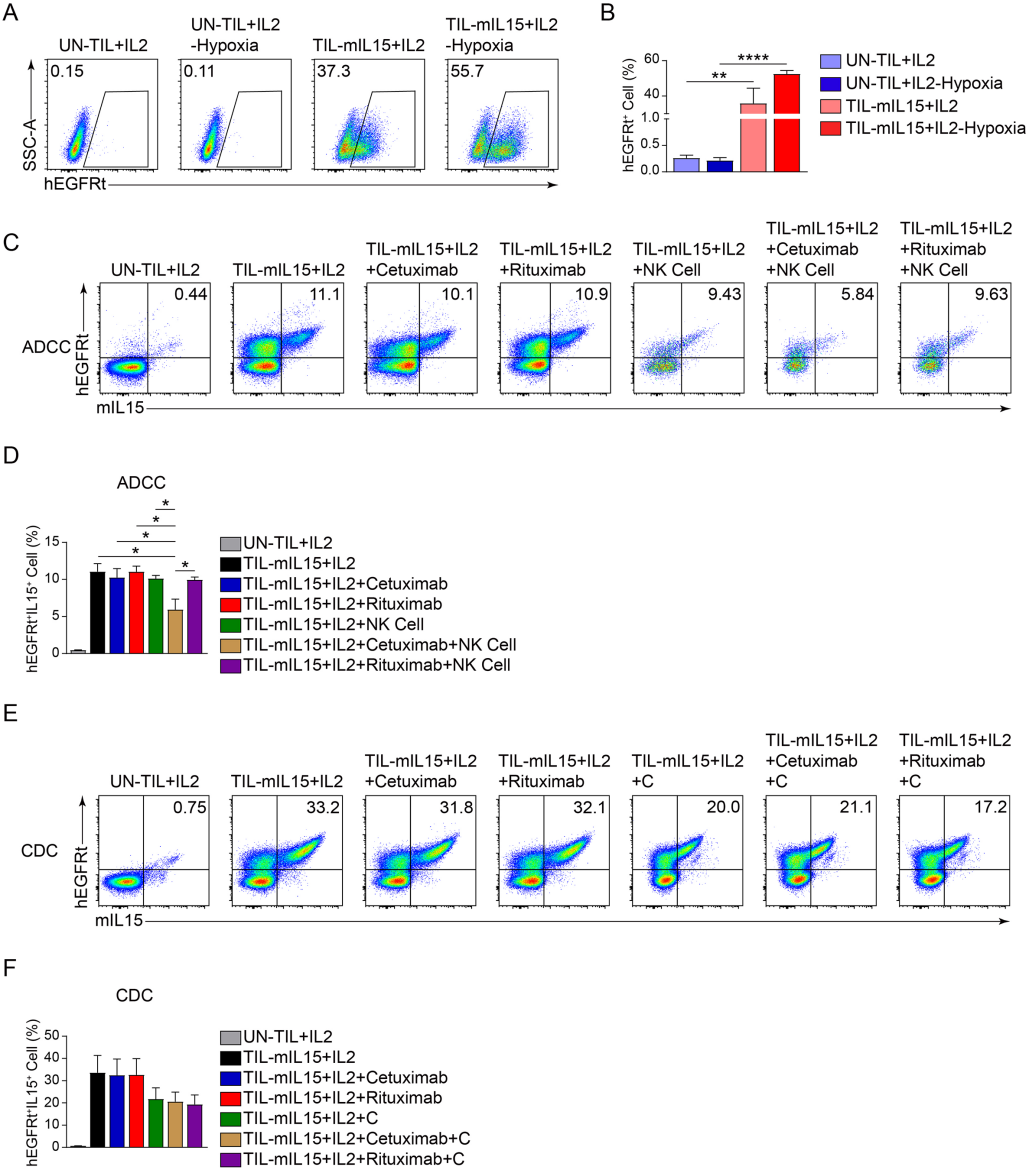
Effect of hEGFRt molecule. **(A)** Representative flow cytometry plot of hEGFRt^+^ cells at 5 d after pre-REP TIL transduction. **(B)** Statistical plot showing the percentage of hEGFRt^+^ cells (n=3). **(C)** ADCC: After 14 d of expansion based on the K562-mIL21-4-1BBL cell culture platform, TIL-mIL15+IL2 cells were cultured with cetuximab, rituximab, and NK cells for 24 h, and flow cytometry was performed to measure proportion of mIL15^+^hEGFRt^+^ cells. **(D)** Statistical plot showing proportion of mIL15^+^hEGFRt^+^ cells (n=3). **(E)** CDC: After 14 d of expansion based on K562-mIL21-4-1BBL cell culture platform, TIL-mIL15+IL2 cells were cultured with cetuximab, rituximab, and complement for 4 h, and flow cytometry was performed to measure the proportion of mIL15^+^hEGFRt^+^ cells. **(F)** Statistical plot showing proportion of mIL15^+^hEGFRt^+^ cells (n=3). Statistical analysis performed using two-tailed unpaired Student’s t-test. Statistical significance defined as follows: *P<0.05; **P<0.01; ****P<0.0001.

Additionally, the hEGFRt molecule was added to the lentiviral vector to enhance the clinical safety of TIL-mIL15 (**Figure 1A**). Apoptosis can be induced in tumor cells with high EGFR expression through ADCC and complement-dependent cytotoxicity mediated by EGFR-targeting antibodies, such as cetuximab (50). Therefore, we designed an experiment using cetuximab to induce apoptosis in TIL-mIL15 cells, to examine the response of hEGFRt-expressing TILs and predicting the response of TIL-mIL15 to cetuximab *in vivo*. After pre-REP TILs were activated with anti-hCD3/hCD28 antibodies, lentiviral transduction was performed for 5 days, and UN-TIL+IL2 cells were used as a control. As shown in **Figures 6A, B**, after UN-TIL+IL2 and TIL-mIL15+IL2 cells were treated with hypoxia for 24 h, the proportion of hEGFRt^+^ cells in the TIL-mIL15+IL2 cells was higher compared to TIL-mIL15+IL2 cells without hypoxia treatment. After expansion for 14 days on the K562-mIL21-4-1BBL cell culture platform, TIL-mIL15+IL2 cells were cultured with cetuximab, rituximab, and NK cells for 24 h. Compared to the other groups, the addition of cetuximab and NK cells led to a decrease in the proportion of mIL15^+^hEGFRt^+^ cells among TIL-mIL15+IL2 cells (**Figures 6C, D**). After expansion for 14 days on the K562-mIL21-4-1BBL cell culture platform, TIL-mIL15+IL2 cells were cultured with cetuximab, rituximab, and complement for 4 h. Compared to the other groups, the addition of cetuximab and complement did not lead to changes in the proportion of mIL15^+^hEGFRt^+^ cells among TIL-mIL15+IL2 cells (**Figures 6E, F**). These results indicated that the combination of hEGFRt expression and cetuximab can induce ADCC in TIL-mIL15 cells. Therefore, if side effects occur during the clinical application of TIL-mIL15 cells, cetuximab injection can induce ADCC, leading to cell apoptosis.

## 4 Discussion

In the mid-1990s, Ratto et al. published a paper on the first TIL-ACT trial in NSCLC, which demonstrated the feasibility of this treatment modality for this type of malignancy (51). In a recently disclosed phase II multi-center study, lifileucel (LN-145) showed good efficacy and safety in patients with metastatic NSCLC, with an objective response rate of 21.4% (52). We overexpressed mIL15 on TILs using a lentiviral infection system to investigate the effects of mIL15 on expansion, stemness, and cytokine secretion of TILs. Our findings revealed that in a culture system without IL2, mIL15 expression can still able to promote TIL proliferation and maintain cell viability at above 90%.

The success of TIL-ACT mainly relies on two factors: the specific recognition by TILs for different antigens of autologous tumors and the capacity of TILs for tumor cell lysis. Response to treatment is positively correlated with the total number of CD8^+^ TILs infused (16). In this study, compared with the irradiated PBMCs cell culture platform, CD8^+^ T cells were enriched in TIL-mIL15 cells based on the K562-mIL21-4-1BBL cell culture platform. Based on the K562-mIL21-4-1BBL cell culture platform, the proportion of mIL15^+^ cells among TIL-mIL15-IL2 cells was significantly increased. Therefore, K562-mIL21-4-1BBL cells used as feeder cells were superior to irradiated PBMCs in the expansion of TIL-mIL15 cells.

While the infusion of mutation-reactive T cells appears crucial to the success of the TIL response, the lineage differentiation status of T cells may play a critical role. The *in vivo* persistence and expansion of adoptively transferred tumor-specific T cells are vital to attaining durable clinical responses (53). In a cohort of melanoma patients treated with TILs, CD8^+^ T cells with stem-like surface markers were associated with tumor cell lysis and durable responses (48). In fact, the majority of mutation-reactive T cells are terminally differentiated and not associated with clinical benefits. However, a small proportion of mutation-reactive T cells were detected in the CD39^-^CD69^-^ stem-like state, and these cells were associated with durable remission, whereas CD39^+^CD69^+^ cells were terminally differentiated and associated with reduced TIL persistence (48). In this study, based on the K562-mIL21-4-1BBL cell culture platform, compared to the UN-TIL+IL2 and TIL-mIL15+IL2 groups, the proportion and absolute number of CD39^-^CD69^-^ cells in the TIL-mIL15-IL2 group were significantly higher, whereas the proportion and absolute number of CD39^+^CD69^+^ cells were significantly lower. The memory stem-like T cell marker *TCF7* has been reported to be highly expressed in CD39^-^CD69^-^TILs (48). Our findings are consistent with those of previous reports. According to RNA-Seq data, TIL-mIL15-IL2 cells expanded based on the K562-mIL21-4-1BBL cell culture platform showed higher expression level of *TCF7* compared to UN-TIL+IL2 and TIL-mIL15+IL2 cells. Therefore, we predict that mIL15-expressing TILs will exhibit greater persistence and expansion potential.

Another undesirable effect of T cell *ex vivo* expansion is the generation of exhausted T cells, which can restrict their persistence and anti-tumor efficacy *in vivo* (54). We observed that, based on the K562-mIL21-4-1BBL cell culture platform, the expression levels of inhibitory receptors LAG3, TIGIT, and TIM3 were significantly reduced in TIL-mIL15-IL2 cells compared to UN-TIL+IL2 and TIL-mIL15+IL2 cells, whereas the expression of the immune checkpoint PD-1 did not differ significantly. The reduced expression levels of these inhibitory receptors promoted *ex vivo* expansion and persistence of TILs.

IFN-γ plays a key role in the anti-tumor immune response. IFN-γ can enhance the antigen presentation and expression of costimulatory molecules on dendritic cells and macrophages, enhance the cytotoxicity of NK cells, and increase the production of chemokines (e.g., IP-10) (55). More importantly, IFN-γ can act directly on tumor cells to enhance immune recognition by upregulating major histocompatibility complex (MHC) class I expression and promoting cell apoptosis (56). However, we found that based on the K562-mIL21-4-1BBL cell culture platform, TIL-mIL15+IL2 and TIL-mIL15-IL2 cells showed significantly decreased IFN-γ secretion compared to UN-TIL+IL2 cells. Previous report revealed that CAR-T cells overexpressing IL15 did not exhibit enhanced effector cytokine production or cytotoxic activity in the short-term killing assay, but showed an enhanced capacity for killing in the continuous killing assay (57). In this study, killing assay showed that the killing capacity of TIL-mIL15+IL2 and TIL-mIL15-IL2 cells was similar (in HCC827 cells) or slightly improved (in NCI-H520) compared with UN-TIL+IL2 cells. These data suggested reduced IFN-γ and GZMB secretion did not affect the killing capacity of TIL-mIL15+IL2 and TIL-mIL15-IL2 cells. This may be due to different mechanisms of T cell activation since anti-CD2/CD3/CD28-conjugated T cell activation magnetic beads were used in IFN-γ and GZMB secretion assay while lung cancer tumor cells were used in killing assay.

As IL15 is a T-cell growth cytokine, excessive and uncontrolled IL15 expression may result in clinical side effects. Therefore, we added hEGFRt as a safety switch to regulate potentially unnecessary and uncontrolled expansion. It was found that under the action of cetuximab and NK cell-mediated ADCC, the proportion of hEGFRt^+^IL15^+^ cells among TIL-mIL15 cells decreased significantly. Hence, the design of this protocol can enhance the safety of TIL-mIL15 for clinical application.

## 5 Conclusion

The production of modified TIL cells, especially in response to T cell exhaustion and the clinical side effects of IL2, has made the development of improvements in this treatment method more urgent. We proposed a modification strategy for TILs such that the TIL-mIL15 cells can automatically sense the hypoxic and potentially avoid possible clinical risks of widespread expression. A hEGFRt switch was also added to further ensure its safety. Our data indicated that this strategy generates more memory stem-like T cells and fewer exhausted T cells *in vitro*, and hence it is expected to bring about greater clinical benefits. The feasibility of this approach was demonstrated using lung cancer TILs, which can be applied to other cancers.

## Data Availability

The original contributions presented in the study are included in the article/**Supplementary Materials**, further inquiries can be directed to the corresponding author/s.

## Supplementary material

Supplementary Figure 1 mIL15 regulates the expression levels of inhibitory receptors. Detection of LAG3, PD-1, TIGIT, and TIM3 expression on CD8^+^ T cells in the UN-TIL+IL2, TIL-mIL15+IL2 and TIL-mIL15-IL2 groups after 14 d of expansion on irradiated PBMCs and K562-mIL21-4-1BBL cell culture platforms.
