# Supplementary figures and images for: Effect of hypoxia-induced mIL15 expression on expansion and memory progenitor stem-like TILs in vitro
Source: Front Immunol. 2024 Nov 22;15:1450245. doi: 10.3389/fimmu.2024.1450245 (PMC11621077; doi:10.3389/fimmu.2024.1450245)

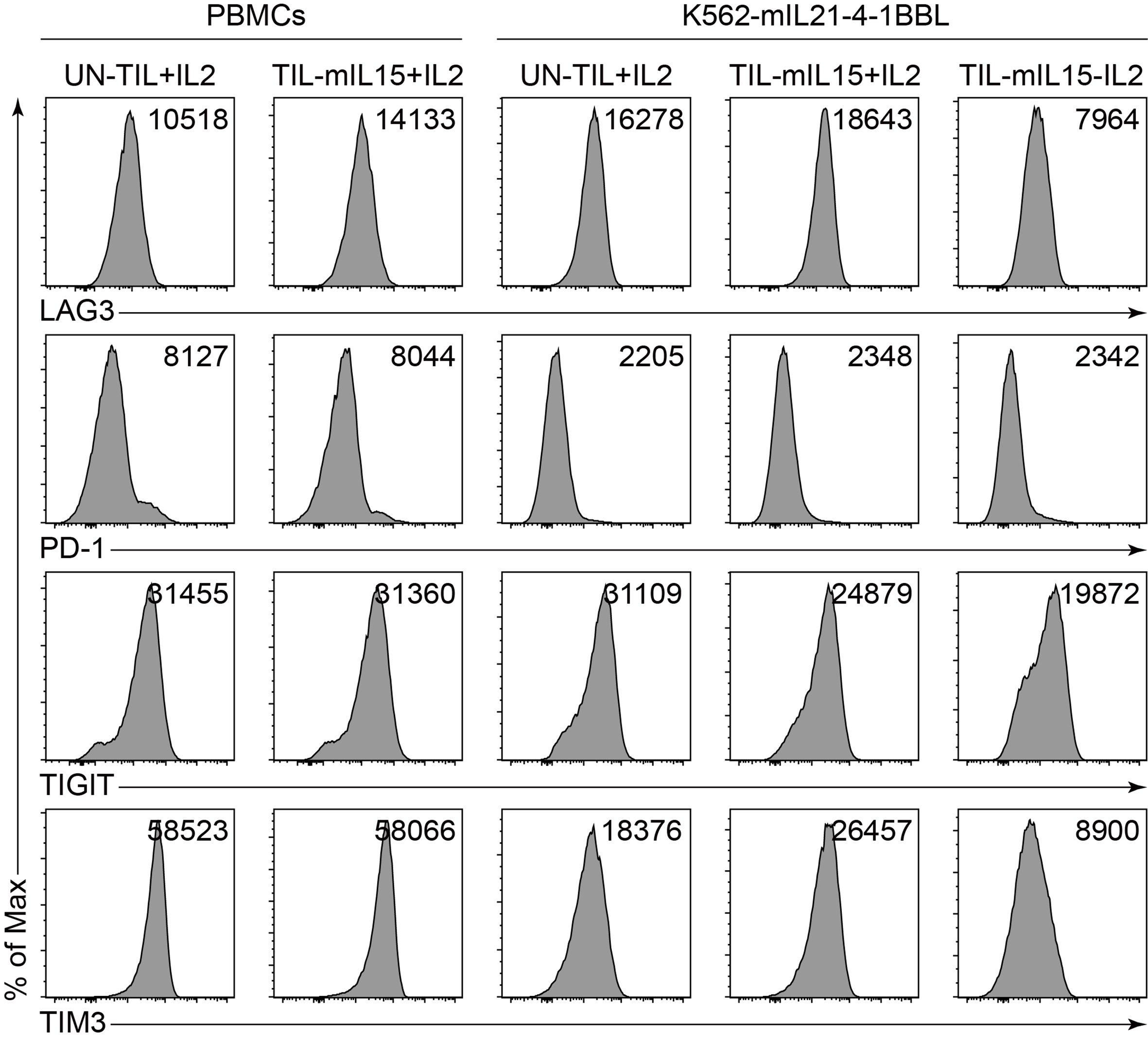

Supplement: Supplementary Figure 1 — mIL15 regulates the expression levels of inhibitory receptors. Detection of LAG3, PD-1, TIGIT, and TIM3 expression on CD8+ T cells in the UN-TIL+IL2, TIL-mIL15+IL2 and TIL-mIL15-IL2 groups after 14 d of expansion on irradiated PBMCs and K562-mIL21-4-1BBL cell culture platforms. [file Image1.tif]
